# A cluster-randomised feasibility trial of a children’s weight management programme: the Child weigHt mANaGement for Ethnically diverse communities (CHANGE) study

**DOI:** 10.1186/s40814-018-0373-6

**Published:** 2018-11-26

**Authors:** Miranda Pallan, Kiya L. Hurley, Tania Griffin, Emma Lancashire, Jacqueline Blissett, Emma Frew, Paramjit Gill, Karla Hemming, Louise Jackson, Kate Jolly, Eleanor McGee, Jayne Parry, Janice L. Thompson, Peymane Adab

**Affiliations:** 1 Institute of Applied Health Research, Murray Learning Centre, University of Birmingham, Edgbaston, Birmingham, B15 2TT UK; 2 School of Life and Health Sciences, Aston University, Aston Triangle, Birmingham, B4 7ET UK; 3 Warwick Medical School, University of Warwick, Coventry, CV4 7AL UK; 4 Birmingham Community Healthcare NHS Trust, 1 Priestley Wharf, Holt Street, Birmingham, B7 4BN UK; 5 School of Sport, Exercise and Rehabilitation Sciences, University of Birmingham, Edgbaston, Birmingham, B15 2TT UK

**Keywords:** Child, Overweight, Obesity, Cultural adaptation, Feasibility studies

## Abstract

**Background:**

Community-based programmes for children with excess weight are widely available, but few have been developed to meet the needs of culturally diverse populations. We adapted an existing children’s weight management programme, focusing on Pakistani and Bangladeshi communities. We report the evaluation of this programme to assess feasibility of programme delivery, acceptability of the programme to participants from diverse communities, and feasibility of methods to inform a future trial.

**Methods:**

A cluster-randomised feasibility trial was undertaken in a large UK city. Children’s weight management programmes (*n* = 24) were randomised to be delivered as the adapted or the standard programme (2:1 ratio). Routine data on participant attendance (*n* = 243) at the sessions were used to estimate the proportion of families completing the adapted and standard programmes (to indicate programme acceptability). Families planning to attend the programmes were recruited to participate in the feasibility study (*n* = 92). Outcome data were collected from children and parents at baseline, end of programme, and 6 months post-programme. A subsample (*n* = 24) of those attending the adapted programme participated in interviews to gain their views of the content and delivery and assess programme acceptability. Feasibility of programme delivery was assessed through observation and consultation with facilitators, and data on costs were collected.

**Results:**

The proportion of Pakistani and Bangladeshi families and families of all ethnicities completing the adapted programme was similar: 78.8% (95% CI 64.8–88.2%) and 76.3% (95% CI 67.0–83.6%) respectively. OR for completion of adapted vs. standard programme was 2.40 (95% CI 1.32–4.34, *p* = 0.004). The programme was feasible to deliver with some refinements, and participant interview data showed that the programme was well received. Study participant recruitment was successful, but attrition was high (35% at 6 months). Data collection was mostly feasible, but participant burden was high. Data collection on cost of programme delivery was feasible, but costs to families were more challenging to capture.

**Conclusions:**

This culturally adapted programme was feasible to deliver and highly acceptable to participants, with increased completion rates compared with the standard programme. Consideration should be given to a future trial to evaluate its clinical and cost-effectiveness.

**Trial registration:**

ISRCTN81798055, registered: 13/05/2014

**Electronic supplementary material:**

The online version of this article (10.1186/s40814-018-0373-6) contains supplementary material, which is available to authorized users.

## Background

Childhood obesity is a global issue, with rising prevalence across high-, middle-, and low-income countries [1]. In the UK, obesity prevalence in children aged 11 years is 20% [2] but varies by ethnicity, with a disproportionate increase in obesity in South Asian children across the middle childhood years (increasing from 10 to 25% between the ages of 5 and 11 years) [2]. This is of importance as the relationship between adiposity and some cardiometabolic risk factors is stronger in South Asian children, compared with the general UK population [3].

Numerous behavioural, family-based interventions targeting children with excess weight in the primary school age group (4–11 years) have been developed and delivered in a range of settings (hospitals, primary care, and community settings [4]). In the past 15 years, many community-based child weight management programmes have been delivered within the UK and other high-income countries. Synthesis of data from trials evaluating these interventions has shown that such programmes lead to BMI *z*-score reductions of around 0.1 at 6 months after programme completion in primary school-aged children [5, 6]. This is clinically significant as even small reductions in BMI *z*-score in children are associated with lower cardiovascular risk [7]. Programme features associated with effective weight loss include elements to address both diet and physical activity, behaviour change techniques, and the involvement of parents [5, 8]. In addition, there is evidence to suggest that better programme attendance is related to increased weight loss [9]. However, there is little evidence of sustained effects from these programmes.

A further issue is that programmes have mainly been evaluated in homogenous cultural groups and have not been developed to address the cultural diversity that is apparent in many communities. The cultural contexts within which families operate are interwoven with other multiple influences on children’s diet and physical activity behaviours [10], and there is increasing recognition of the need to adapt health promotion strategies to address the different cultural contexts within our diverse communities [11]. One study reports an evaluation of a programme developed in the USA and delivered within an ethnically diverse community in the UK. No cultural adaptation of the programme was undertaken, and the evaluation did not show any positive effect on BMI *z*-score, compared with the control group [12]. There is some evidence to suggest that minority ethnic communities engage less well with children’s weight management programmes [13, 14], but this is not consistent [15].

In response to the highlighted need for health promotion programmes to meet the needs of the diverse communities in the UK [11], we culturally adapted an existing children’s weight management programme that was being delivered to families of children with excess weight in a large, superdiverse UK city (Birmingham; population = ~ 1.1 million). The programme, First Steps, delivered across Birmingham since 2010, had shown that among families who completed the programme the effect on children’s BMI *z*-score was comparable to that reported in previous research. Although initial take up of the programme was similar across all ethnic groups, the proportion of families dropping out of the programme was higher in Pakistani and Bangladeshi families (40% of these families completing the programme compared with 65% of families overall—unpublished, routinely collected service data). Therefore, we undertook a theoretically informed cultural adaptation process, which focused primarily on families from Pakistani and Bangladeshi communities. These communities are distinct but also have many similarities, including the strong influence of the Islamic faith on beliefs and behaviour, the central role of mosques (places of worship) for social interaction, and the relative socioeconomic disadvantage that is present within these communities, which influences norms and behaviours [16]. However, from the outset, we recognised that the concept of ‘ethnicity’ is an over simplification, and diversity within communities arises from the dynamic interplay of factors linked to migration [17]. Therefore, our overall aim was to develop a flexible and responsive programme that would be suitable for all families within culturally diverse communities, and we accounted for this in our adaptation process, which is described in a separate report [18]. In brief, we undertook an adaptation process guided by the Behaviour Change Wheel framework [19] and the Typology of cultural adaptations and health promotion programme theory proposed by Liu and colleagues [11]. Adaptations were made at both surface and deep structural levels.

In this paper, we report the findings of a feasibility trial of the culturally adapted programme: the Child weigHt mANaGement for Ethnically diverse communities (CHANGE) study. The primary aim was to assess the acceptability of the programme to families attending and the feasibility of programme delivery. A secondary aim was to assess the feasibility of trial methods, recruitment, and data collection to inform the design of a future randomised evaluation of the clinical and cost-effectiveness of the programme.

## Methods

### Design and randomisation

A two-arm cluster-randomised controlled trial design was employed with the weight management programmes as the cluster units (as described in the published protocol [20]). This design enabled the primary outcome of completion of the adapted programme to be estimated and compared with completion of the standard programme. The cluster design also enabled participants to attend the programme nearest to them, which would not have been the case if an individually randomised design had been undertaken. All programmes delivered in Birmingham between September 2015 and April 2016 (*n* = 24) were randomised to the intervention (adapted programme) or comparator (standard programme) arms with a 2:1 ratio (to give a more precise estimate of completion in the intervention arm). The families of all eligible children referred to the weight management service were invited to attend the programme most convenient for them. Eligibility criteria for the service were: child aged 4–11 years with excess weight (BMI over the 91st centile of the UK 1990 growth reference charts [21]); resident in Birmingham; and able to participate in a group programme.

Randomisation of the programmes was conducted in STATA 13 (StatCorp, Texas, USA) by a member of the Birmingham Primary Care Clinical Research & Trials Unit (AR) before the start of the feasibility study. Four half-termly cycles of programme delivery (four adapted programmes and two standard programmes per school half-term) were planned; therefore, randomisation was stratified by delivery cycle. Allocations were communicated to the service providers so they could plan programme delivery, but were concealed from the research team and programme participants.

### Participant recruitment and follow-up

To assess programme completion, routinely collected, anonymised attendance data from all programme participants were used. For evaluation of other outcomes related to acceptability of the programme, recruitment strategy, trial processes, and data collection procedures, families who booked to attend a programme during the study period were invited to participate in the CHANGE feasibility trial. All families enrolled on the programme were eligible to participate in the trial. Families were invited to participate by letter, followed up by a telephone call in their preferred language. If they agreed to participate, an appointment at their home was arranged to gain written consent and undertake baseline assessments. Written consent was obtained from all participating parents. Children were asked for written assent if they were 8 or more years and verbal assent if younger.

Baseline data collection took place from families consenting to participate in the study before they attended a programme (T0). Follow-up home appointments were made at programme end (T1) and 6 months later (T2) to collect outcome data. All participating families were given a £10 shopping voucher at T1 in recognition of their contribution to study measures.

### Intervention

The adapted intervention programme was delivered as six 90 min sessions at weekly intervals, with parents and their children attending all sessions. A higher proportion of the adapted programmes were delivered on weekend days compared with the standard programmes. The adapted programme (reported in detail in Additional file 1) was more interactive than the standard programme, involving several activities each week. The focus of the programme was on promoting healthy behaviours (rather than weight loss, which was the focus of the standard programme), and session content covered healthy eating, physical activity, making changes to behaviour, and activities to develop skills in these areas. Weekly goals were set and reviewed. Two facilitators delivered the adapted programme during the study period, with assistance from a third staff member. Prior to delivery, facilitators attended two training sessions and a facilitator manual was provided.

### Comparator

The standard weight management programme was delivered in the comparator arm by a different facilitator to avoid contamination. The programme was run as five to seven weekly sessions (depending on the length of the school half-term); the first and last were 90 min in duration and attended by parents and children, and the remaining sessions were 60 min and attended by parents only. Details of the comparator programme are given in Additional file 2.

### Assessment of programme acceptability and feasibility of delivery

#### Programme completion

The primary outcome was estimation of the proportion of Pakistani and Bangladeshi families completing the adapted programme, using routinely collected, anonymised attendance data from families attending the programmes. Completion was pragmatically defined as attending ≥ 60% of sessions as we recognised that other commitments or unexpected events may prevent families from attending all sessions, despite their intention to do so. Additional outcomes were completion rates of all families in both programmes, and odds ratios for completing the adapted versus the standard programme in Pakistani and Bangladeshi, and in all families attending.

#### Observation of programme delivery and facilitator feedback

Observations of sessions were undertaken by the research team throughout the intervention period to evaluate programme implementation and participant engagement. In addition, during the first cycle of delivery, the facilitators were asked to feedback on their experience of delivery after each session. This feedback, together with information from observations in the first delivery cycle, was used to further refine the adapted programme for the subsequent three delivery cycles.

#### Interviews with programme facilitators, parents, and children

At the end of the study intervention period, semi-structured interviews were conducted with the facilitators and parents and children (aged ≥ 8 years) who attended the programme. Interview schedules were developed to guide discussions. We aimed to recruit approximately 15 parents and 10 children, with approximately 50% Pakistani/Bangladeshi participants, and a mix of completing and non-completing parents. We developed interview schedules to explore experiences of programme delivery and participation and whether participants were able to make changes to their health-behaviour following the programme. Interviews were undertaken in the participants’ preferred languages by research team members (TG and KLH-white British; MA-Pakistani). Face-to-face interviews were undertaken in participants’ homes, and where this was not possible, telephone interviews were conducted. We obtained written consent (facilitators and parents) or assent (children) from all participants, and they each received a £10 shopping voucher in addition to that given for the main study.

Interviews were audio-recorded, translated into English (where required), and transcribed verbatim. A sample was back translated by an independent researcher to check for accuracy. Data analysis was guided by the Framework approach [22] and conducted by two researchers (TG and KLH). Transcript coding was undertaken using NVivo 10 (QSR International Pty Ltd); the researchers independently coded a sample of transcripts then discussed and agreed a final coding framework, which was applied to all transcripts.

### Measurement of health-related outcomes

Data on a range of health-related outcome measures were collected from participating children and parents at the three time points (baseline (T0), programme end (T1), and at 6-months (T2)) by trained researchers using standardised assessment protocols. Questionnaire-based outcome measures were administered in the participants’ preferred language. The outcomes and their assessment methods are shown in Table 1.

**Table 1:** Outcome data collected from participating children, parents, and other family members

Assessment	Data collection method	Data processing
Child assessments		
Sex, date of birth, postcode	Obtained from weight management service records, verified by parent/child	Home postcodes mapped to Index of Multiple Deprivation (IMD) 2015 scores [34], which were categorised into quintiles using nationally derived quintile cut offs
Ethnicity, religion, language	Reported by parent/carer	
Height	Marsden Weighing Group © Leicester Height Measure HM-250P (two measures taken, with a third measure if > 4 mm difference; two closest measures averaged)	BMI calculated (kg/m^2^); age- and sex-specific *z*-scores derived for BMI, percentage body fat, and waist circumference using the relevant UK reference data [21, 35, 36]
Weight and percentage body fat	TANITA® BC-420MA body composition scales (light clothing, no shoes, empty bladder)	
Waist circumference	Lufkin® W606 PM flexible steel tape measure (two measures taken; with a third measure if > 4 mm difference; two closest measures averaged)	
Pubertal status	Simplified visual assessment of breast development in girls, facial hair in boys (based on the Tanner scale [37]). Parent report whether girls had started menstruating	
Objective 7-day physical activity record	Wrist-worn GeneActiv© (Activinsights, Cambs, UK) or waist-worn Actigraph GT3X + © (ActiGraph, Pensacola, FL) on non-dominant side of body for 7 days	
Health-related quality of life	Pediatric Quality of Life Inventory™ (PedsQL) [38, 39] (aged 5–7 or 8–12 years; self-report)	Scales converted to 0–100 point scales, with higher scores indicating better quality of life; total score and subscale scores calculated (physical, emotional, social and school functioning)
	The Child Health Utility 9D (CHU 9D) [40–42]—a preference based measure of health-related quality of life for use in children, allowing calculation of QALYs^a^	
Body image questionnaire	Figure Rating Scale (adapted for use in multi-ethnic populations) [43, 44]	Body dissatisfaction scores derived by subtracting ‘ideal self’ from ‘self’ score (range of scores: − 8 to + 8; 0 indicates body satisfaction, negative values indicate child would like to be smaller, positive values indicate child would like to be larger)
Child dietary patterns	Children’s Dietary Questionnaire [45] (completed by parent/carer)—28-item questionnaire that measures intake patterns of a variety of healthy and unhealthy foods; adapted for use in the local population	Scores calculated for intake of fruit and vegetables; dairy; sugar-sweetened beverages; and non-core foods
Parent assessments		
Ethnicity, religion, language, place of birth and when moved to UK, age when left full time education, highest educational qualification, employment status	Self-reported	
Family diet and activity habits	Family Nutrition and Physical activity survey [46]	Total score calculated
Authoritative parenting style	Authoritative parenting dimension of the Parenting Styles and Dimensions questionnaire [47]	Subscale score from 1 to 5 calculated
Parental self-efficacy	Parental Locus of Control scale [48]	Subscale score from 1 to 5 calculated
Parental feeding practices	Comprehensive Feeding Practices Questionnaire [49] (9 subscales included)	Subscale scores from 1 to 5 calculated (child control, encouraging balance and variety, environment, modelling, monitoring, restriction for health, restriction for weight control, teaching about nutrition, and involvement)
Height, weight, % body fat	As for child	BMI calculated (kg/m^2^)
Assessments with other family members		
Date of birth	Self-reported (or proxy reported by parent for younger children)	
Height, weight, % body fat	As for child	BMI calculated (kg/m^2^)

^a^*QALY* quality-adjusted life year

To assess children’s physical activity, two types of triaxial accelerometer were used and compared: GeneActiv© (Activinsights, Cambs, UK—wrist-worn) and Actigraph GT3X+© (ActiGraph, Pensacola, FL—waist-worn). The purpose of this was to assess which accelerometer had highest compliance (defined as wearing monitor for > 10 h on at least 1 day) and data completeness. We planned to use the GeneActiv on 75% of participants and GT3X+ monitors on the remainder. At each time point, children wore the accelerometer for 7 days and wore the same accelerometer type at all time points. Both devices were set to record at 100 Hz in 60 s epochs. Data were analysed using the GGIR package v1.4 developed in R v3.3.1 (R Foundation for Statistical Computing, Vienna, Austria) through the University of Birmingham BlueBEAR High Performance Computing service [23]. A valid day’s wear was defined as > 10 h wear time in a 24-h period. Device and wear position-specific cut-points were used to estimate minutes spent in moderate, vigorous, and moderate to vigorous activity as previously reported in the literature [24–26].

We tested the feasibility of collecting anthropometric data (height, weight, and percentage body fat) on parents and other family members (see Table 1) as this data would enable evaluation of the wider family impact of the adapted programme in a future trial.

### Costs associated with the intervention

We explored methods to measure costs from a societal perspective, including intervention-specific costs, parent productivity costs, associated childcare costs, and changes to the family’s weekly food bill. For the intervention and the comparator programmes, methods were developed to capture the resource use and costs associated with programme material production, venue hire, programme management, and staff costs. Staff training costs were also estimated for the intervention programme. For families, costs linked to time spent participating in the programme and any changes in behaviour resulting from attending the programme (including child care costs and changes to the family food bill) were estimated through a survey administered at the final session.

### Sample size

From routine service data, the mean number of Pakistani/Bangladeshi families in each programme group (cluster) was 5. Delivery of 16 adapted programmes was planned in the intervention arm. Therefore, assuming an intra-cluster correlation coefficient of 0.05, 16 clusters and a mean cluster size of 5 allowed estimation of the proportion of Pakistani and Bangladeshi families completing the programme to within 26% precision (variance expected under individual randomisation was inflated to account for clusters and varying cluster size [27]). To evaluate recruitment and data collection feasibility, we planned to recruit at least 80 families to participate in the study. As the focus of the programme adaptation was on Pakistani and Bangladeshi families, we aimed to purposefully recruit 48 families (60% of the sample) from these communities.

### Statistical analysis

Statistical analysis was conducted in STATA 13 (Texas, USA). Using routinely collected service data, we estimated the primary outcome of the proportion of Pakistani and Bangladeshi families completing the adapted programme, adjusting the 95% CI using robust standard errors to account for the effect of clustering. We used the same methods to estimate the proportion of families of all ethnicities completing in each arm, and of Pakistani and Bangladeshi families in the comparator arm. To estimate the odds ratios for completion in the adapted vs. standard programme, we developed mixed effects logistic regression models, adjusted for clustering, and then further adjusted for child sex and age. We compared routinely collected child data on study participants and programme participants not taking part in the study to assess representativeness of the study sample.

We summarised baseline characteristics of study participants by study arm using mean (SD), median (IQR), or proportions, as appropriate. We calculated the proportion of participants with complete data for each health-related outcome measure at each time point to assess data completeness.

We undertook an exploratory analysis to assess costs associated with the adapted programme, and inform methods for economic evaluation in a future trial. We assessed incremental costs of the adapted programme by measuring the resource use associated with both the standard and adapted elements of the programme and focussing on the difference in costs between the two elements.

## Results

### Programme participation and completion

**Fig. 1:**
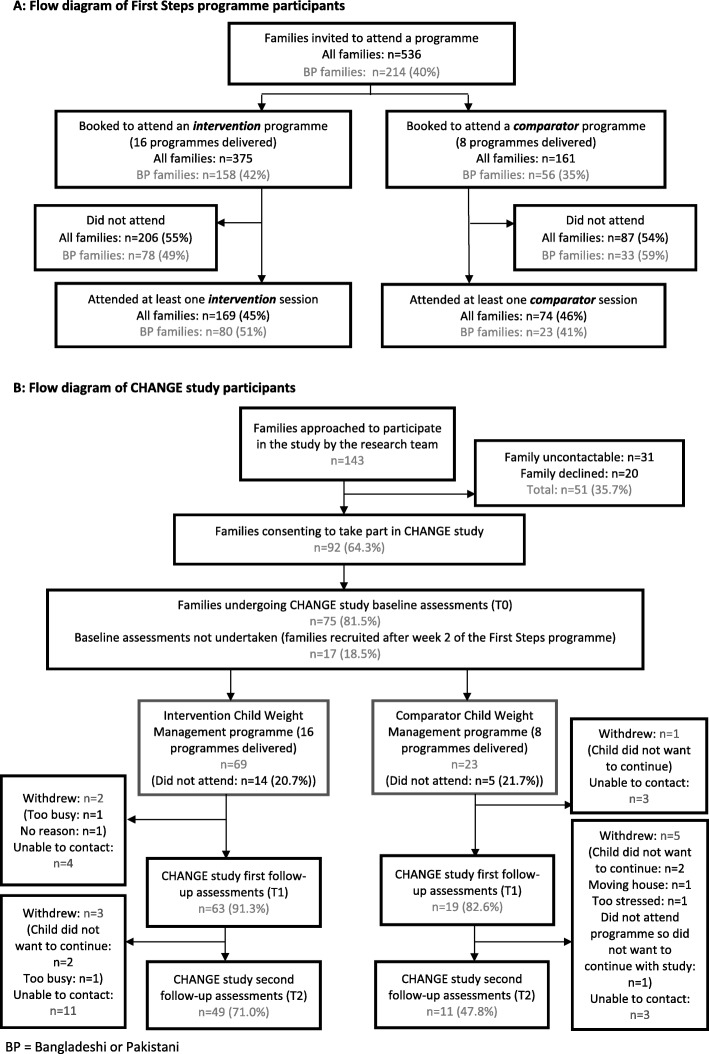
Flow of programme and study participants. **a** Flow diagram of First Steps programme participants. **b** Flow diagram of CHANGE study participants

From September 2015 to April 2016, 536 families (40% Pakistani or Bangladeshi) were invited to attend a programme following referral through multiple routes (e.g. General Practitioner, self-referral, referral as part of a national child BMI surveillance programme [28]). Of these, 243 (45%) attended at least one session. Figure 1a shows the flow of participants through the adapted and standard programmes.

The proportions of families completing the adapted and standard programmes are shown in Table 2. Completion rates were higher for the adapted, compared with the standard programme (age, sex, and cluster-adjusted OR 2.40, 95% CI 1.32–4.34; *p* = 0.004). For the adapted programme, completion was similar for Pakistani and Bangladeshi families, 78.8% (95% CI 64.8–88.2%) and all families, 76.3% (95% CI 67.0–83.6%).

**Table 2:** Proportion of programme participants completing the adapted and standard programmes, and odds ratios (adapted: standard) for programme completion

	Adapted programme			Standard programme				Model 1^a^			Model 2^b, c^	
	A1 (*n*)	COM (*n*)	% (95% CI^a^)	A1 (*n*)	COM (*n*)	% (95% CI^a^)	*n*	OR (95% CI)	*p* value	*n*	OR (95% CI)	*p* value
BP families	80	63	78.8 (64.8, 88.2)	23	14	60.9 (48.5, 72.0)	103	2.38 (0.88, 6.43)	0.09	101	2.49 (0.91, 6.80)	0.07
Non-BP families	83	62	74.7 (65.0, 82.4)	45	26	57.8 (40.9, 73.4)	128	2.15 (1.00, 4.66)	0.05	128	2.13 (0.94, 4.80)	0.07
All families ^d^	169	129	76.3 (67.0, 83.6)	74	43	58.1 (46.5, 68.8)	243	2.36 (1.26, 4.42)	0.007	241	2.40 (1.32, 4.34)	0.004

*BP* Bangladeshi and Pakistani, *A1* number of families who attended at least once, *COM* number of families who completed the programme (attended > 60%), OR odds ratio

^a^Adjusted for clustering

^b^Additionally adjusted for child sex and child age at start of the programme

^c^Two families not included in the model as not data available for child’s sex or age

^d^Ethnicity unknown for 12 families

### Study participants

Of 143 families approached by the study team between September 2015 and April 2016, 92 (64.3%) consented to participate in study assessments. Due to logistical difficulties (e.g. short timeframe from the booking of a family onto a programme to them commencing the programme, and family and researcher availability for home visits), we only obtained baseline measures at T0 from 75 participants (81.5%). The remaining 17 families participated in data collection at T1, and their baseline data were recorded as missing. We collected follow-up data from 82 families at T1 (end of programme; 89.1%) and 60 families at T2 (6 months post intervention 65.2%). Attrition was high (35%), with 11 (12%) families actively withdrawing and 21 (23%) lost to follow-up, despite multiple attempts to contact them. There was greater attrition in the standard programme arm (52.2% vs. 29.0% in the adapted programme arm). Participant characteristics were similar in those followed up compared with those lost to follow-up (data not shown). Nineteen families (20.7%) did not attend any programme sessions (14 (20.3%) in the adapted programme and 5 (21.7%) in the standard programme; Fig. 1b).

Overall, child age, sex, ethnicity, and baseline BMI *z*-score were similar in those consenting compared with those who did not consent to participate (data not shown). Almost half of the study participants were of Pakistani or Bangladeshi ethnicity. Baseline characteristics of participating children by study arm are shown in Table 3.

**Table 3:** Study participant baseline characteristics

	Intervention (adapted programme)	Comparator (standard programme)	Total
	*n* = 69	*n* = 23	*n* = 92
	*n* (%^a^) or mean (SD)	*n* (%^a^) or mean (SD)	*n* (%^a^) or mean (SD)
Sex of the child (*n* = 92)			
Male	32 (46.4)	12 (52.2)	44 (47.8)
Female	37 (53.6)	11 (47.8)	48 (52.2)
Age at start of course (years; *n* = 92)	10 (2.0)	10 (2.0)	10 (2.0)
Ethnicity (*n* = 91)			
White	8 (11.8)	6 (26.1)	14 (15.4)
Black	6 (8.8)	3 (13.0)	9 (9.9)
Pakistani/Bangladeshi	36 (52.9)	8 (34.8)	44 (48.4)
Indian	5 (7.4)	2 (8.7)	7 (7.7)
Mixed/other ethnicities	13 (19.1)	4 (17.4)	17 (18.7)
IMD quintile (*n* = 90)			
1 (most deprived)	53 (79.1)	18 (78.3)	71 (78.9)
2	9 (13.4)	1 (4.4)	10 (11.1)
3	2 (3.0)	4 (17.4)	6 (6.7)
4	3 (4.5)	0 (0.0)	3 (3.3)
5 (least deprived)	0 (0.0)	0 (0.0)	0 (0.0)
First language (*n* = 89)			
English	58 (86.6)	19 (86.4)	77 (86.5)
Urdu	4 (6.0)	0 (0.0)	4 (4.5)
Mirpuri	1 (1.5)	1 (4.6)	2 (2.3)
Sylheti	1 (1.5)	0 (0.0)	1 (1.1)
Bengali	1 (1.5)	1 (4.6)	2 (2.3)
Other	2 (3.0)	1 (4.6)	3 (3.4)
Religious identity (*n* = 82)			
Muslim	42 (65.6)	8 (44.4)	50 (61.0)
Sikh	1 (1.6)	2 (11.1)	3 (3.7)
Hindu	1 (1.6)	1 (5.6)	2 (2.4)
Christian	7 (10.9)	5 (27.8)	12 (14.6)
Other	1 (1.6)	0 (0.0)	1 (1.2)
No religion	12 (18.8)	2 (11.1)	14 (17.1)
Body mass index *z*-score (*n* = 75)	2.5 (0.6)	2.7 (0.7)	2.5 (0.6)
Body fat percentage *z*-score (*n* = 73)	2.2 (0.4)	2.3 (0.4)	2.2 (0.4)
Waist circumference *z*-score (*n* = 69)	2.8 (0.7)	3.1 (0.5)	2.9 (0.6)
Puberty commenced (*n* = 65)	13 (28.3)	8 (42.1)	21 (32.3)
Average acceleration^b^ (*n* = 64, SVMg; mg)	32.1 (14.7)	32.0 (12.1)	32.1 (14.0)
Moderate to vigorous physical activity^b^ (*n* = 64, min/24 h)	14.5 (11.2)	11.0 (22.7)	13.6 (12.2)
Pediatric Quality of Life Inventory score^b^ (*n* = 71)	76.1 (23.9)	70.63 (28.1)	75.2 (24.2)
Physical functioning score^b^ (*n* = 72)	81.25 (15.7)	81.25 (21.9)	81.3 (17.2)
Emotional functioning score^b^ (*n* = 74)	80.0 (40.0)	75.0 (30.0)	77.5 (35.0)
Social functioning score^b^ (*n* = 75)	75.0 (30.0)	60.0 (40.0)	70.0 (40.0)
School functioning score^b^ (*n* = 75)	75.0 (25.0)	70.0 (45.0)	75.0 (30.0)
Body dissatisfaction score^b^ (*n* = 73)	2.0 (2.0)	2.0 (2.0)	2.0 (2.0)
Child Health Utility score (*n* = 75)	0.85 (0.12)	0.89 (0.09)	0.86 (0.11)
Children’s Dietary Questionnaire			
Fruit and vegetable score (*n* = 67)	5.9 (2.9)	4.6 (3.0)	5.5 (3.0)
Dairy score^b^ (*n* = 61)	2.0 (2.0)	1.0 (2.0)	2.0 (2.0)
Sugar-sweetened beverages score^b^ (*n* = 68)	1.3 (1.9)	1.4 (2.1)	1.3 (1.9)
Non-core foods score^b^ (*n* = 63)	2.4 (1.5)	2.0 (2,6)	2.3 (1.6)
Family Nutrition and Physical Activity score (*n* = 53)	57.4 (5.5)	54.8 (7.2)	56.6 (6.1)
Authoritative parenting score (*n* = 47)	4.3 (0.6)	4.2 (0.3)	4.3 (0.6)
Parenting efficacy score (*n* = 50)	2.2 (0.6)	2.1 (0.6)	2.2 (0.6)
Parent feeding practices scores			
Child control (*n* = 42)	1.7 (0.7)	2.2 (0.7)	1.8 (0.8)
Encouraging balance and variety^b^ (*n* = 42)	3.5 (0.8)	3.3 (1.5)	3.5 (1.0)
Environment^b^ (*n* = 42)	3.0 (1.3)	3.0 (1.8)	3.0 (1.3)
Modelling (*n* = 42)	2.6 (0.9)	2.4 (1.4)	2.5 (1.0)
Monitoring (*n* = 42)	2.7 (0.8)	2.9 (1.1)	2.7 (0.9)
Restriction for health^b^ (*n* = 42)	3.0 (1.0)	3.5 (1.0)	3.3 (1.0)
Restriction for weight control (*n* = 42)	2.5 (0.7)	2.0 (1.0)	2.4 (0.8)
Teaching about nutrition (*n* = 42)	2.8 (0.8)	2.7 (0.8)	2.8 (0.8)
Involvement (*n* = 42)	2.2 (0.7)	2.4 (0.7)	2.2 (0.7)

^a^Percentages may not sum to 100 due to rounding

^b^Summary statistic = median (IQR)

### Programme observation and facilitator feedback

The CHANGE research team observed delivery of 12 of the sessions across nine adapted programmes, including at least one of each of the six programme sessions, and both facilitators. Feedback was received from the facilitators after each session for the first cycle of programme delivery, after which the facilitator manual and intervention materials were finalised. Table 4 shows the issues identified through observation and feedback, and the resulting refinements made to the programme. In general, the facilitator feedback was very positive, although they highlighted some specific issues, particularly relating to the healthy eating and food preparation sessions (weeks 2 and 5). Some issues identified through observation and facilitator feedback, such as disruptions caused by families arriving late and the logistical challenges of large group sizes, could not be easily addressed. The presence of interpreters for non-English speaking participants worked well.

**Table 4:** Programme observation and facilitator feedback, resulting programme refinements

Programme session	Issue identified	Changes made/actions taken
Week 2—healthy eating	Visual aids used in the standard programme that had deliberately not been included in the adapted programme were used by facilitators	Researchers rehearsed the week 2 session plan with the facilitators and reminded them not to use the visual aids from the standard programme
	Too much material to deliver within 90 min	The number of activities in the session was reduced to ensure the key nutrition messages were delivered
	Some of the nutrition messages were not clearly delivered and participants appeared to be confused on occasions	Content was streamlined to ensure more focus on the core nutrition and healthy living messages. Additional notes on key nutritional concepts were included in the facilitator’s manual
Week 4—physical activity	Delivery of this session did not require 90 min	Facilitators were encouraged to reiterate nutrition messages in this session
Week 5—give it a go	Challenging to deliver with only one facilitator, especially with larger group sizes	Extra facilitator provided for this session. A plan for setting up the materials in advance of the session was developed. Participant worksheets were simplified to enable more families to work through them with less facilitator input
	The ‘make a healthy snack’ activity was too messy	The number of healthy snack making options was reduced from four to two, retaining the least messy options
	The recipe planning activity did not work well	Recipe planning was removed from the session and the group provided with recipes to take home to try
Week 6—review and celebrate	Facilitators felt uncomfortable awarding particular participants the ‘star achiever’ certificate	All children received a completion certificate and the ‘star achiever’ certificate was removed
All sessions	Weekly goal setting and review elements were sometimes missed or rushed and not covered adequately. Participants often arrived late, interrupting the flow of the session	Week 2 was streamlined to allow more time for review and setting of goals. The importance of goal setting and review as a key behaviour change technique was explained to the facilitators and further highlighted in the manual

After programme delivery was completed, both facilitators were interviewed (facilitator 1 = female, Pakistani, delivered 12 adapted programmes, interviewed via telephone; facilitator 2 = female, white British, delivered 4 adapted programmes, interviewed face-to-face). Interviews with 16 parents (10 mothers and 6 fathers; 11 completers) and 9 children (all completers, aged 10–12 years) who attended the adapted programme were conducted. Six parents and two children were of Pakistani/Bangladeshi ethnicity. Three parents were interviewed in another language (two in Urdu, one in Mirpuri).

### Retrospective views of the programme from facilitators, parents, and children

The experiences of all interviewees were generally very positive. Parents reported behaviour change within their families, even if they did not complete the programme, and facilitators valued the programme flexibility. Attending at the weekends generally worked well for families, although logistical issues were a problem for some. Parents felt that there was value in children attending all sessions so that they were exposed to health messages directly from an alternative authoritative figure. Parents and facilitators felt that the wide age range of children attending was problematic, particularly keeping the younger children engaged. All interviewees welcomed the interactive activities and peer support, and parents and children would have liked more physical activity and food preparation elements. In contrast, the facilitators felt the adaptations made to the programme resulted in insufficient content to develop knowledge and skills relating to nutrition. One of the facilitators also felt that the focus on promoting healthy behaviour rather than weight loss in the adapted programme was unhelpful. This viewpoint was not evident among the other interviewees. The website developed to support the programme was well received by the facilitators, but rarely used by programme participants. Quotes to illustrate these findings are presented in Table 5.

**Table 5:** Views of parents, children and facilitators following programme attendance/delivery

Programme element	Views of parents, children, and facilitators after end of programme
General experiences	‘It was a nice refreshing change to see that we could facilitate rather than actually talk, erm, some of the families to death at times so it was, it was I would say nice.’ (Facilitator 1)
	‘the whole thing it was delivered so softly it was just about making sensible choices, informed choices, you know, and I think that we got all the tools that we needed to do that, you know, we were told everything and the way it was delivered was superb, I can’t say that enough it was just from start to finish it was a really good course. Er, and yeah we’ve continued doing it and we’ve made changes and we’re continuing to make changes’ (P2148, Father, Black, completer)
	‘I think all of it was because I could see her weight and I could see what I could do as a parent to help her and all my family. And my husband made a few changes in his diet as well’ (P2079, Mother, Pakistani, non-completer)
Facilitator guide	‘And for me to have my guide, my facilitator guide, so I’m sitting there with that one guide constantly and making notes and thinking about it um I like that as well, that’s a big help.’ (Facilitator 2)
Programme timing, attendance and barriers to attendance	‘It does make a massive difference because the family can come [on Saturdays], the whole family can come whereas during the week you know even some adults find it difficult to take that hour and a half you know they’re working’ (Facilitator 2)
	‘Because of getting from school to go it was – and because going to college and stuff, it’s kind of – was kind of impossible to attend every session.’ (P2055, Mother, Black, non-completer)
Children attending	‘having the kids involved was such a big plus for us because like I said, there were lots of reasons why we’re, it was, you know, it was good to see them with their parent and what their relationship was like and act with them’ (Facilitator 1)
	‘It was actually quite useful because most of the time children won’t listen to parents, but when they see a professional explaining they take on board.’ (P2092, Mother, Black, completer)
	‘when you’ve got four and five year olds there they’re not interested because they’re four and five, whereas when they’re kind of six and seven they can sit and talk to you and listen’ (Facilitator 2)
	‘it was about an hour and a half, and I think it was just too long for some of the little ones to sit and listen.’ (P2115a, Mother, White, completer)
Focus on healthy behaviours	‘we always veered away from the words weight, overweight, and it was healthy lifestyle which is fine but the results at the end anecdotally I don’t think were as good in that regard.’ (Facilitator 2)
Healthy eating/nutritional knowledge	‘the only thing that I really struggled with was week two [healthy eating session] to actually get that, all that information across and it didn’t really, then it kind of went against what we were trying to do, which was giving them a chance to sort of interact with us, because there was so much’ (Facilitator 1)
	‘They explained quite well, I mean what to eat and what should be avoided. Although I already knew about this but it is common when we attend such events we always learn some new information, so this helped me a lot.’ (P2017, Mother, Pakistani, non-completer)
Physical activity	‘Well I would have liked to um, in the course I would like to like be more active, like run around and stuff’ (C2063, Child, Pakistani, completer)
	‘I think that’s my biggest frustration about the course itself is that we’ve moved too far down the road to saying physical activity is key’ (Facilitator 2)
Interactive activities	‘it was quite fun altogether because we had to go round the room and we had to find the different foods. Then you had to find out how much they were labelled in, er how much sugar and fats are there in there.’ C2025, Child, Pakistani, completer)
	‘Yeah but I thought there’d be more like activities for the kids and stuff, I think they only had activities on one day and the other time they had, that’s when they had to make snacks, so the rest of the time they were sitting there’ (P2124, Mother, Black, completer)
Peer support	‘There was a better I would say, erm, social environment between I would say the participants because we gave them I think more opportunities as well though in this course.’ (Facilitator 1)
	‘It was nice to be around other people that had the same like issues with weight management stuff with their children, because the support was good, as in, you know, to be around other parents and, you know, just generally when you talk to the parents and stuff’ (P2112, Mother, Pakistani, completer)
	‘The things that I enjoyed was like, I got to make new friends and everything.’ (C2091, Child, Indian, completer)
Programme website	‘I think it [the website] is great because we’d always been asking for a resource that we could give them um anyway, so I think it’s a great idea and I do like it, and I think it’s easy to get around’ (Facilitator 2)
	‘we did use it every now and again just to look at food, the sugar content and the fat content because of the items on the site’ (P2148, Father, Black, completer)

### Feasibility of data collection

Data collection in the participant’s home proved to be challenging. Despite appointment reminders, there were several occasions where researchers found that the family were not home, and faced subsequent difficulties in rearranging the appointment. The overburdening of participants was also an issue, with a median time for a data collection visit of 60 min. As a result, we modified data collection from parents so that some questionnaire data (sociodemographic information, Children’s Dietary Questionnaire, Family Nutrition and Physical Activity questionnaire) were collected during the visit and other questionnaires (parenting style, parental self-efficacy, and parental feeding practices) were completed by parents after the visit and returned by post. Data collection visits were longer for non-English speaking participants, as researchers needed to verbally translate all questionnaires. A further challenge was concealment of participants’ study arm from the researcher. At T1 and T2 visits, the study arm was sometimes revealed to the researchers through general conversation.

For the participants who provided data at each time point, the proportion providing data for each health-related outcome measure (and the mean/median value) is shown in Additional file 3. Height and weight measures were completed for all children. Of the anthropometric measures, waist circumference had the most missing data at each time point (15–28%). Researchers reported that this was most often due to child refusal. Child-completed questionnaires (PedsQL, Figure Rating Scale, and CHU 9D) were generally well completed (87–100%). Parent-completed questionnaires that were administered during the data collection visit had moderate to good completion rates (62–97%), but those that could be returned by post were less well completed (43–67%). Usable physical activity data were available for 85% at baseline, 82% at T1, and 73% at T2. Physical activity monitor compliance was compared for GeneActiv and Actigraph GT3X+ (Additional file 4). In general, compliance was higher for the GeneActiv. Collection of anthropometric data from parents and other family members proved problematic, particularly as the family members who consented to be measured at each time point were often not the same. Therefore, collection of these data was not feasible.

### Costs associated with the intervention programme

Data were collected on setup costs for the adapted and standard programmes, including staff training and equipment costs. Total setup costs were £178 for the standard and £940 for the adapted programme; additional costs were related to staff training and visual aids used in the sessions. We also measured the delivery costs, focusing on the difference between the two programmes, which included the provision of adapted materials, venue hire, and staffing costs. For both programmes, average resource use was estimated based on an assumed full attendance of families at each session (i.e. materials prepared based on attendance of all families at all sessions). The incremental cost of materials for the adapted programme, compared with the standard programme, per family was £3.09. Regarding venue hire and staffing, the incremental costs of the adapted programme were £287.70 per session and £27.24 per family attending. Table 6 summarises the additional costs associated with the adapted programme.

**Table 6:** Total incremental cost for the adapted programme compared with the standard programme

Description	Average incremental cost(per session)	Average incremental cost (per attender)
	£	£
Materials	41.08	3.09
Additional staffing costs	70.2	6.65
Additional venue hire costs	217.5	20.59
Average incremental cost	328.78	30.33

An end of programme survey was completed by 96 participants. Sixty-six percent of these completed the question on what they would be doing if not attending the programme and 53% the question about changes to their weekly food bill since starting the programme. Only 4% stated they would have been in paid employment if they had not been at the programme and no respondents had to pay for dependants while they attended. More than half of respondents (54%) reported a change to their food bill (44% noticing an increase and 56% a decrease).

## Discussion

### Feasibility and acceptability of the adapted programme

The adapted programme was successfully delivered and acceptable to participating families. A key indicator of acceptability was the proportion of families completing the programme. This was 76% overall (79% of Pakistani and Bangladeshi families and 75% of other families). Families attending the adapted programme were nearly 2.5 times as likely to complete compared with families attending the standard programme. These findings concur with the interview data from participants in which they articulate the high acceptability of the programme. These completion rates also compare favourably to reported completion for other community-based children’s weight management programmes [29–31].

The structure of weekly sessions over an average of 6 weeks was acceptable to participants and feasible in terms of cost and delivery, although it had higher staffing and venue costs compared with the standard programme. The facilitators were enthusiastic about delivering the programme, and participants and facilitators valued the flexible, interactive, and supportive nature of the programme. These elements were explicitly identified in the theoretical adaptation process that we undertook [18]. No divergent views across different ethnic groups emerged from the participant interviews. One facilitator felt that the de-emphasis on weight loss in the adapted programme may have lessened its impact, but this view was not shared, and the need to reframe messages in children’s weight management programmes to prevent weight stigma has been highlighted [32]. The increase in physical activity content for the adapted programme also split opinion as participants would have liked even more physical activity but facilitators felt that this overshadowed the nutrition content. Participants also expressed a wish for more interactive activities, such as the food preparation activity.

### Feasibility of trial design, recruitment, and data collection

The cluster-randomised design enabled direct comparison of completion of the adapted programme with that of the standard programme and also allowed participants to attend the most convenient programme for them. However, in a future trial to evaluate effectiveness, consideration needs to be given to the comparator arm. Given the small reported effect sizes of community weight management programmes [5, 6], the difference in effect one would expect if comparing the adapted programme with a comparator programme may be very small, and so an adequately powered trial would not be feasible. In addition, the landscape of provision of children’s community weight management programmes in the UK is changing (i.e. much less service provision than in previous years), such that no provision is ‘standard’ in many areas. Therefore, a more appropriate trial design would be an individually randomised trial with no active programme in the comparator arm.

Although we achieved our recruitment target of 80, we experienced logistical challenges in collecting baseline data between the point of recruitment and the family attending the first programme session. Design of a future trial would need to ensure that baseline data are collected from participants before intervention commencement. This could be achieved through more streamlined recruitment processes across the service provider and research team and a longer time interval between the booking and commencement of a programme.

Participant attrition was a major issue, with 35% loss at 6 months. This is in line with other studies, although there is a large variation (1–42% [5]). The high attrition in this study may in part be explained by the setting: Birmingham has a highly mobile, young population [33]. An additional problem was the differential attrition in study arms (29% in the intervention arm vs. 52% in the comparator arm), which would make interpretation of outcomes difficult in a trial. This high attrition occurred despite attempts to minimise it (e.g. home visits for data collection, text and letter reminders, etc.). Further, incentives for participants at 6 months should be considered in a future trial, and attrition needs to be accounted for in the sample size calculation. In addition, imputation or other appropriate methods to account for missing follow-up data should be considered so that an intention-to-treat analysis approach could be undertaken.

Collection of outcome data through home visits was acceptable, but resource intensive. Difficulty in concealment of study arm allocation from researchers was an issue, but could be overcome in a future trial by separation of the research staff undertaking the outcome measures and the core research team responsible for the delivery of the study. The amount of outcome data collected overburdened participating parents, which may have affected participant attrition. Therefore, in a future trial, outcome data would need to be streamlined, focusing on a few key outcome measures.

Methods were successfully developed to measure the costs associated with delivering the adapted programme, which could be employed in a future trial. Data capture on costs to families of attending a programme was less successful, due to the low return of questionnaires at the final programme session. The response may be improved by collecting this data alongside the study outcome data. The core outcome data that would be used in a future cost-effectiveness or cost-utility analysis were collected successfully (BMI *z*-score, CHU 9D). However, it was not feasible to collect the data to capture impact of the intervention on the wider family.

### Strengths and limitations

The cluster-randomised design enabled evaluation of the feasibility and acceptability of the adapted programme, and the processes and methods required for a future clinical and cost-effectiveness evaluation. The qualitative evaluation methods enabled us to explore the engagement of programme facilitators and participants and subsequent change in behaviours. We were also able to use this information to refine the adapted programme. We tested recruitment and follow-up to 6 months, and the collection of cost and outcome data, and gained valuable information to inform a future trial.

Although the programme was adapted primarily to increase acceptability to Pakistani and Bangladeshi families, we evaluated its acceptability in an ethnically diverse population. A key adaptation was the flexibility of delivery and responsiveness of the programme, so it was important to assess how it was received in a diverse population. A particular strength of the study was the inclusion of non-English speaking participants, which would also be important in a future trial.

One limitation, which was not the focus of this feasibility study, was initial programme attendance. In both study arms, just over 50% of families booked to attend the programmes did not attend any sessions. Even among CHANGE study participants, 20% did not attend any programme sessions. This is of concern for the future provision of children’s weight management services, but was beyond the scope of this study. A further limitation is that we have not tested the acceptability of individual randomisation or allocation to a control arm where no intervention is received.

## Conclusions

Through this feasibility trial, we have shown that a community-based children’s weight management programme, adapted to be flexible and responsive enough to meet the needs of all families in diverse communities, was feasible to deliver and highly acceptable to participating families. This was demonstrated through increased retention of families in the adapted programme compared with the standard programme. The study also highlights further areas to address in the design of a future trial. In particular, attrition would need to be minimised and accounted for in the trial sample size. Given the high acceptability and feasibility of delivery of the adapted children’s weight management programme, consideration should be given to conducting a clinical and cost-effectiveness trial.

## Additional files

Additional file 1: The CHANGE study adapted children’s weight management programme: Template for Intervention Description and Replication (TIDieR) checklist. (DOCX 23 kb)

Additional file 2: The CHANGE study comparator children’s weight management programme: Template for Intervention Description and Replication (TIDieR) checklist). (DOCX 25 kb)

Additional file 3: Data provision and mean/median for each outcome measure at each time point. (DOCX 16 kb)

Additional file 4: Data provision and median scores at each time point for physical activity monitors. (DOCX 12 kb)

## Abbreviations

BMI: Body mass index
BP: Bangladeshi or Pakistani
CHANGE: Child weigHt mANaGement for Ethnically diverse communities (study acronym)
CHU 9D: Child Health Utility 9D
CI: Confidence interval
IMD: Index of multiple deprivation
IQR: Interquartile range
OR: Odds ratio
PedsQL: Pediatric Quality of Life Inventory
QALY: Quality-adjusted life year
SD: Standard deviation
T0: Time point 0 (baseline)
T1: Time point 1 (end of programme)
T2: Time point 2 (6 months post-programme)
