# Supplementary material for: A cluster-randomised feasibility trial of a children’s weight management programme: the Child weigHt mANaGement for Ethnically diverse communities (CHANGE) study
Source: Pilot Feasibility Stud. 2018 Nov 26;4:175. doi: 10.1186/s40814-018-0373-6 (PMC6260774; doi:10.1186/s40814-018-0373-6)
Supplement: Supplementary file 2 — The CHANGE study comparator children’s weight management programme: Template for Intervention Description and Replication (TIDieR) checklist). (DOCX 25 kb) [file 40814_2018_373_MOESM2_ESM.docx]

**The CHANGE study comparator children's weight management programme: Template for Intervention Description and Replication (TIDieR) checklist)**

| **1: Name** | ***First Steps*** |
| --- | --- |
| **2: Why** | The standard First Steps programme was developed by Birmingham Community Healthcare NHS Trust (BCHCT), based on research evidence and their own local experiences, and has been delivered in Birmingham since 2010. |
| **3& 4: Materials and Procedures** | Families referred to the children's weight management service are sent a letter inviting them to attend their nearest programme. Families receiving an initial invitation letter also have a follow up phone call in their preferred language.  Parents attend all sessions and children attend the first and last sessions. Non-English speaking participants are accompanied by an interpreter.  Flip charts and handouts (including Change4Life handouts (<https://www.nhs.uk/change4life>)) are used to assist in delivery of the programme. Games and props are also used for some of the interactive activities. Paper folders to put handouts in are given to the families.  Aims and outlines of the seven programme sessions are as follows:  *Week 1: Introductory session*  Aims   - Introduce the programme, find out about the expectations of the group and how to manage them - Inform families about the content and structure of the First Steps Programme - Establish group ground rules for the course and ensure that the families understand the commitment required - Collect data through the completion of questionnaires and height/weight data - Introduce the families to the sugar and fat game   Outline   1. Introduce programme 2. Icebreaker activity: participants say their name and a food they like. They then write their expectations of the course on a sticky note 3. Introduce topics covered on the course, relate them to the expectations of the group. Share examples of success stories 4. Flipchart activity: participants suggest ground rules 5. Height and weight measurements conducted with children, parents asked to complete questionnaires on eating and exercise behaviours 6. Activity: fat and sugar quiz 7. Set weekly target: swap snack to a healthier alternative   *Week 2: Eatwell plate^a^ and 5 A Day*  Aims   - Review the families' swap snack targets - Give an overview of the key healthy eating messages the Eatwell plate represents - Enable participants to review their own diet in relation the Eatwell plate, and make suggestions for changes - Consider ways to increase fruit and vegetable intake - Build upon the healthy snack swaps and encourage a more balanced diet   Outline   1. Recap on programme aims and session plan 2. Recap on fat and sugar quiz, checking what participants have learnt, and discuss snack swaps the families have made 3. Activity: Eatwell plate - participants place foods on the relevant segment of the Eatwell plate, facilitator then discusses each segment 4. Activity: participants complete a blank Eatwell sheet with what they ate yesterday, and are given a further sheet for their child to complete at home 5. Introduce home activity: 5 A day fruit and vegetable record sheet to be completed with their family every day for a week   *Week 3: My Personal Health Plan & The Parent’s Role*  Aims   - Review the Eatwell plate and 5 a day record chart - Introduce the importance of making a plan to change - Start my personal health plan which will be built upon each week - Introduce behaviour change diary to use with personal health plan to help monitor change - Introduce the idea of ‘healthy rewards’ to maintain change - Identify helpful techniques that parents/carers may use in creating a supportive and healthy home environment, focusing on food and physical activity   Outline   1. Recap the last session's key messages and review the home-completed Eatwell plate and 5 A Day record 2. Introduce the purpose of the session: thinking about how to make changes within the family 3. Flipchart exercise: ask 'why do you think it is important to have a plan to make changes?' 4. Completion of the Personal Health Plan (PHP) handout and behaviour change diary 5. Introduce home activity: create a new PHP with their child / family and use the behaviour change diary to record the changes they make 6. Give participants the healthy rewards handout and explain the importance of not using foods as rewards 7. Flipchart exercise: mindmap ideas about positive parenting 8. Discuss role modelling using a case study 9. Discuss the importance of rules and boundaries 10. Introduce home activity: family to discuss and agree on ground rules relating to food and physical activity 11. Discuss behaviour management techniques 12. Give handouts on importance of sleep and tips for parents   *Week 4: Regular meals and snacks*  Aims   - Explain the importance of regular healthy meals and snacks - Discuss which snacks are healthy - Introduce strategies for parents if a child is saying they are still hungry   Outline   1. Recap on last session, and review family rules, use of PHPs and behaviour change diaries 2. Introduce the importance of regular meals and limiting snacks; discuss the '3 + 2' rule 3. Flipchart exercise: ask 'what are the benefits of regular eating?' and discuss 4. Snacks: discuss what a snack is and ask parents what they give their children for snacks. Record on flipchart, discuss which are healthy options and get group to think of more healthy snacks 5. Discuss how parents cope with children demanding snacks 6. Importance of breakfast 7. Activity: is my child hungry? 8. Introduce home activity: complete 3+2 diary with their child 9. Ask participants to complete a new PHP and behaviour change diary   *Week 5: Portion sizes and food labelling*  Aims   - Encourage participants to consider the influence of inappropriate portion sizes - Demonstrate general guideline amounts for the portion sizes of selected foods - Help participants acknowledge their own eating habits - Explain the nutrition information on food labels.   Outline   1. Recap on 3+2 message and ask parents to share their PHPs and diaries 2. Introduce the importance of food quantity as well as food types 3. Portion size tips: discuss and give handout 4. Introduce the 'Y plate' and the 'Steps guide to healthy food in the right proportions plate' as tools to help serve the right proportion of foods and the appropriate amount 5. Give handout on 'Me Size Meals' and 'My Hand, My Portion Size' 6. Food labelling: what are the difficulties the group have? 7. Discuss key areas of food labelling: ingredients list; nutrition information table; traffic lights. 8. Give food labelling rules hand out and explain importance of comparing nutrient content per 100g 9. Activity: food label quiz and review of food labels brought in by participants 10. Introduce home activity: foods in cupboard worksheet to complete with child 11. Ask participants to complete a new PHP and behaviour change diary   *Week 6: Physical Activity and takeaways*  Aims   - Help participants increase levels of physical activity - Help participants recognise some of the nutritional consequences of ‘fast food’ - Help participants make healthier choices when eating away from home - Help participants cook their own dishes at home   Outline   1. Recap on last session and review food cupboard worksheet, PHP and behaviour change diary 2. Introduce the importance of physical activity and the concept of energy balance (draw seesaw to illustrate) 3. Flipchart exercise: other benefits of physical activity and how much physical activity should children do 4. Give 'Why exercise is important' handout and discuss recommendations for physical and sedentary activity for children 5. Activity: physical activity card game (how much energy do we use when doing certain activities?) 6. Discuss how to build physical activity into everyday life, give handout on low cost activities 7. Highlight local physical activity opportunities and give Birmingham Physical Activity Information pack 8. Discuss ways of motivating children to do physical activity 9. Introduce home activity: weekly activity chart to complete with child 10. Flipchart exercise: participants' thoughts on eating out and takeaways 11. Discuss consequence of too much fat - use 'blocked artery' picture and fat model to illustrate 12. Flipchart exercise: healthy alternatives to takeaways 13. Give handouts on the facts about takeaways and healthy alternatives 14. Discuss the cost of takeaways in comparison to home cooking 15. Introduce home activity: handout on healthy recipes as alternatives to takeaways - participants to try a recipe and take a photo   *Week 7: Reflection, maintaining behaviour and planning ahead*  Aims   - Give an overview of previous sessions - Help participants recognise their achievements and strengths - Help participants devise a plan for maintaining changes in the future - Provide coping strategies for when they lose their way - For the participant to leave the course feeling supported and confident they can continue their good work in the coming months   Outline   1. Welcome and congratulate participants on completing the programme 2. Recap on physical activity and takeaway messages and review home activities - award prize for best home cooked meal as an alternative to takeaway 3. Recap on all topics covered in the programme and help the group review and reflect on the changes they have made 4. Give 'When things don't go according to plan' handout 5. Conduct height and weight measurements with the children 6. Ask families to complete physical activity, eating habits and evaluation questionnaires 7. Activity: ask families to formulate an action plan and answer the 'Looking Forward' questions 8. Thank everyone for participating and remind them that they will be invited to a follow up event in three months |
| **5: Who provided** | A facilitator employed by the service provider organisation (Birmingham Community Healthcare NHS Trust) delivered the standard First Steps programme (a different facilitator to those delivering the adapted programme). He had experience of delivering the programme since its introduction in 2010. A second employee of the service provider organisation attended the first and last sessions to assist in collecting height and weight data. |
| **6: How** | The programme is designed to be delivered to a group of families (optimum group size is 10 families). |
| **7: Where** | The programme is designed to be delivered in local community venues (e.g. community centres or primary schools). |
| **8: When and how much** | The programme is delivered through seven 1 hour sessions over seven weeks. Programmes run either on week days in school time (75% of programmes) or on Saturdays (25% of programmes). Programmes are delivered to coincide with school term times. |
| **9: Tailoring** | Families are asked to keep behaviour change diaries which are reviewed by the facilitators. There is also provision for families to attend before the group session starts so they can have one-to-one time with the facilitator. |
| **10: Modifications** | When there are short school terms the programme content is combined and delivered over five or six weeks. |
| ^a^Eatwell plate is a pictorial guide produced by the UK Food Standards Agency which shows the proportions and types of foods that are required for a healthy and balanced diet | |
