# Supplementary material for: A cluster-randomised feasibility trial of a children’s weight management programme: the Child weigHt mANaGement for Ethnically diverse communities (CHANGE) study
Source: Pilot Feasibility Stud. 2018 Nov 26;4:175. doi: 10.1186/s40814-018-0373-6 (PMC6260774; doi:10.1186/s40814-018-0373-6)
Supplement: Supplementary file 3 — Data provision and mean/median for each outcome measure at each time point. (DOCX 16 kb) [file 40814_2018_373_MOESM3_ESM.docx]

**Data provision and mean/median for each outcome measure at each time point**

|  | **T0 (n=75)** | | **T1 (n=82)** | | **T2 (n=60)** | |
| --- | --- | --- | --- | --- | --- | --- |
|  | Participants providing data  n (%) | Mean/median | Participants providing data  n (%) | Mean/median | Participants providing data  n (%) | Mean/median |
| BMI z-score (mean (SD)) | 75 (100.00) | 2.52 ( 0.61) | 82 (100.00) | 2.45 ( 0.66) | 60 (100.00) | 2.34 ( 0.60) |
| Body fat percentage z-score (mean (SD)) | 73 ( 97.33) | 2.25 ( 0.41) | 80 ( 97.56) | 2.24 ( 0.46) | 58 ( 96.67) | 2.05 ( 0.54) |
| Waist z-score (mean (SD)) | 64 ( 85.33) | 2.92 ( 0.67) | 68 ( 82.93) | 2.77 ( 0.70) | 43 ( 71.67) | 2.96 ( 0.65) |
| Average acceleration (mg; median (IQR)) | 64 ( 85.33) | 32.07 (14.01) | 67 ( 81.71) | 32.23 (20.75) | 44 ( 73.33) | 34.32 (12.40) |
| Moderate to vigorous physical activity (minutes; median (IQR)) | 64 ( 85.33) | 13.61 (12.18) | 67 ( 81.71) | 16.58 (15.02) | 44 ( 73.33) | 15.93 (13.89) |
| Pediatric Quality of Life Inventory (max=100; median (IQR)) | 71 ( 94.67) | 75.16 (24.22) | 77 ( 93.90) | 83.75  (18.44) | 52 ( 86.67) | 83.67 (21.02) |
| Physical functioning score | 72 ( 96.00) | 81.25 (17.19) | 78 ( 95.12) | 87.50 (25.00) | 56 ( 93.33) | 92.19 (15.63) |
| Emotional functioning score | 74 ( 98.67) | 77.50 (35.00) | 80 ( 97.56) | 87.50 (25.00) | 58 ( 96.67) | 87.50 (35.00) |
| Social functioning score | 75 (100.00) | 70.00 (40.00) | 80 ( 97.56) | 85.00 (30.00) | 58 ( 96.67) | 87.50 (25.00) |
| School functioning score | 75 (100.00) | 75.00 (30.00) | 79 ( 96.34) | 80.00 (25.00) | 60 (100.00) | 80.00 (20.00) |
| Body dissatisfaction score (median (IQR)) | 73 ( 97.33) | 2.00 ( 2.00) | 78 ( 95.12) | 2.00 ( 2.00) | 59 ( 98.33) | 2.00 ( 1.00) |
| Child Health Utility score (max=1.0; mean (SD)) | 75 (100.00) | 0.86 ( 0.11) | 78 ( 95.12) | 0.90 ( 0.09) | 58 ( 96.67) | 0.92 ( 0.06) |
| Children's Dietary Questionnaire |  |  |  |  |  |  |
| Fruit and vegetable score (max=28; mean (SD)) | 67 ( 89.33) | 5.55 ( 3.00) | 75 ( 91.46) | 6.08 ( 2.99) | 58 ( 96.67) | 6.54 ( 3.46) |
| Dairy score (max=15; median (IQR)) | 61 ( 81.33) | 2.00 ( 2.00) | 69 ( 84.15) | 1.00 ( 3.00) | 56 ( 93.33) | 2.00 ( 2.00) |
| Sugar-sweetened beverages score (max=5.9; median (IQR)) | 68 ( 90.67) | 1.29 ( 1.86) | 77 ( 93.90) | 1.29 ( 2.14) | 57 ( 95.00) | 1.29 ( 2.00) |
| Non-core foods score (max=10.3; median (IQR)) | 63 ( 84.00) | 2.29 ( 1.57) | 70 ( 85.37) | 2.00 ( 1.57) | 47 ( 78.33) | 2.57 ( 1.14) |
| Family Nutrition and Physical Activity score (max=80; mean (SD)) | 53 ( 70.67) | 56.60 ( 6.12) | 51 ( 62.20) | 59.60 ( 6.03) | 44 ( 73.33) | 60.25 ( 6.76) |
| Standardised authoritative parenting score (max=5; mean (SD)) | 47 ( 62.67) | 4.26 ( 0.58) | 45 ( 54.88) | 4.34 ( 0.66) | 26 ( 43.33) | 4.27 ( 0.48) |
| Standardised parental efficacy score (max=5; mean (SD)) | 50 ( 66.67) | 2.16 ( 0.60) | 47 ( 57.32) | 2.19 ( 0.63) | 27 ( 45.00) | 2.12 ( 0.68) |
| Standardised parent feeding practices scores (max=5) | |  |  |  |  |  |
| Child control (mean (SD)) | 42 ( 56.00) | 1.81 ( 0.78) | 41 ( 50.00) | 1.80 ( 0.77) | 29 ( 48.33) | 1.54 ( 0.57) |
| Encouraging balance and variety (median (IQR)) | 42 ( 56.00) | 3.50 ( 1.00) | 42 ( 51.22) | 3.50 ( 1.00) | 29 ( 48.33) | 3.50 ( 0.75) |
| Environment (median (IQR)) | 42 ( 56.00) | 3.00 ( 1.25) | 42 ( 51.22) | 3.00 ( 1.00) | 29 ( 48.33) | 3.25 ( 1.50) |
| Modelling (mean (SD)) | 42 ( 56.00) | 2.52 ( 1.04) | 42 ( 51.22) | 2.79 ( 0.76) | 29 ( 48.33) | 2.88 ( 0.71) |
| Monitoring (mean (SD)) | 42 ( 56.00) | 2.72 ( 0.90) | 41 ( 50.00) | 3.01 ( 0.92) | 29 ( 48.33) | 2.99 ( 0.84) |
| Restriction for health (median (IQR)) | 42 ( 56.00) | 3.52 ( 1.00) | 42 ( 51.22) | 3.38 ( 1.00) | 29 ( 48.33) | 3.25 ( 0.75) |
| Restriction for weight control (mean (SD)) | 42 ( 56.00) | 2.36 ( 0.76) | 41 ( 50.00) | 2.41 ( 0.77) | 29 ( 48.33) | 2.13 ( 0.57) |
| Teaching about nutrition (mean (SD)) | 42 ( 56.00) | 2.81 ( 0.80) | 41 ( 50.00) | 3.06 ( 0.67) | 29 ( 48.33) | 2.91 ( 0.78) |
| Involvement (mean (SD)) | 42 ( 56.00) | 2.24 ( 0.70) | 41 ( 50.00) | 2.39 ( 0.85) | 29 ( 48.33) | 2.39 ( 0.91) |
