# Supplementary material for: A cluster-randomised feasibility trial of a children’s weight management programme: the Child weigHt mANaGement for Ethnically diverse communities (CHANGE) study
Source: Pilot Feasibility Stud. 2018 Nov 26;4:175. doi: 10.1186/s40814-018-0373-6 (PMC6260774; doi:10.1186/s40814-018-0373-6)
Supplement: Supplementary file 4 — Data provision and median scores at each time point for physical activity monitors. (DOCX 12 kb) [file 40814_2018_373_MOESM4_ESM.docx]

**Data provision and median scores at each time point for physical activity monitors**

|  | **T0 (n=75)^a^** | | | | **T1 (n=82)^b^** | | | | **T2 (n=60)^c^** | | | |
| --- | --- | --- | --- | --- | --- | --- | --- | --- | --- | --- | --- | --- |
|  | GeneActiv (n=66) | | Actigraph GT3X+ (n=9) | | GeneActiv (n=68) | | Actigraph GT3X+ (n=14) | | GeneActiv (n=50) | | Actigraph GT3X+ (n=10) | |
| Number of extracted files (n (%)) | 58 | (87.9) | 8 | (88.9) | 62 | (93.9) | 11 | (70.0) | 42 | (84.0) | 8 | (80.0) |
| Number with over one day wear recorded (n (%)) | 56 | (84.8) | 8 | (88.9) | 57 | (86.4) | 10 | (62.5) | 36 | (72.0) | 6 | (60.0) |
| Valid days wear if worn (days; median (IQR)) | 5.0 | ( 2.0) | 6.0 | ( 1.5) | 5.0 | ( 2.0) | 4.5 | ( 4.0) | 6.0 | ( 2.0) | 5.0 | ( 2.0) |
| Average acceleration (SVMg; mg; median (IQR)) | 32.9 | (10.8) | 14.7 | ( 3.0) | 33.7 | (16.3) | 13.3 | ( 5.8) | 35.2 | (11.1) | 11.9 | ( 9.9) |
| Moderate-vigorous physical activity (mins/24 hrs; median (IQR)) | 16.0 | (13.3) | 9.9 | ( 5.1) | 18.6 | (15.0) | 8.1 | (12.2) | 16.4 | (13.2) | 4.8 | (10.9) |
| ^a^ 17 children did not have baseline measurements; 6 GeneActivs were not returned and 2 files could not be extracted; 1 Actigraph was not returned | | | | | | | | | | | | |
| ^b^ 5 GeneActivs were not returned and 1 child refused to wear; 3 Actigraphs were not returned | | | | | | | | | | | | |
| ^c^ 7 GeneActivs were not returned and 1 child refused to wear; 1 Actigraph was not returned and 1 child refused to wear | | | | | | | | | | | | |
